# Intravitreal Fluocinolone Acetonide for Diabetic Macular Edema: Long-Term Effect and Structure/Function Correlation

**DOI:** 10.3390/diagnostics12102415

**Published:** 2022-10-06

**Authors:** Angelo Maria Minnella, Martina Maceroni, Claudia Zagami, Elena Quarato, Stanislao Rizzo, Matteo Giarletti, Giorgio Placidi, Benedetto Falsini

**Affiliations:** 1 Institute of Ophthalmology, Università Cattolica del Sacro Cuore, 00168 Rome, Italy; 2 Ophthalmology Unit, Fondazione Policlinico Universitario A. Gemelli—IRCCS, 00168 Rome, Italy

**Keywords:** diabetic macular edema (DME), fluocinolone acetonide (FAc), central macular thickness (CMT), photopic negative response (PhNR)

## Abstract

The long-term effect of intravitreal Fluocinolone acetonide (FAc) on retinal morphology and function in diabetic macular edema (DME) was investigated. Seventeen eyes of twelve consecutive DME patients, treated by intravitreal FAc, were retrospectively evaluated. Retinal morphology was assessed with central macular thickness (CMT). Retinal function was assessed by best-corrected visual acuity (BCVA) and cone b-wave and photopic negative response (PhNR). The main outcome was a mean change in CMT at month 24. The secondary outcomes were changes in cone b-wave and PhNR at month 24. The incidence of adverse events was also recorded. Mean CMT decreased from 406.52 µm (±138.74) at baseline to 310 µm (±130.39) at 24 months (*p* = 0.008). No significant changes in the other parameters were found. At baseline, BCVA and PhNR amplitude were negatively correlated (r = −0.55) with CMT. At the end of follow-up, the change in CMT was negatively correlated with baseline CMT (r = −0.53, *p* = 0.03) and positively correlated with baseline PhNR amplitude (r = 0.58, *p* < 0.01). A significant, long-term reduction in CMT was observed in DME patients after FAc implant. The anti-edema effect tended to be stronger in patients with the poorest baseline retinal morphology (CMT) and function (PhNR). Structure/function correlations might help to characterize the patients who may benefit from this treatment.

## 1. Introduction

Diabetic macular edema (DME) is a manifestation of diabetic retinopathy and is characterized by the breakdown of the blood–retinal barrier and increased vascular permeability, which results in the leakage of fluid and other plasma constituents [1]. In DME, pro-inflammatory cytokines and other inflammatory mediators, including MCP-1, SDF-1, ICAM-1, VCAM-1 and sVAP-1, seem to play a more important role than vascular endothelial growth factor (VEGF), leading to persistent chronic inflammation in the retina, resulting in leucocyte activation, leukostasis and damage to the blood–retinal barrier [2].

Several treatments for DME have been developed and approved for use, such as laser photocoagulation [3], sub-threshold micropulse laser [4,5], intravitreal administration of anti-vascular endothelial growth factor agents (VEGF antagonists) [6], and intravitreal corticosteroids implants [7,8]. Intravitreal corticosteroids are thought to reduce retinal edema through their anti-inflammatory effects, such as inhibition of edema, fibrin deposition, capillary dilation, leukocyte migration, capillary and fibroblast proliferation, collagen deposition, and scar formation [8].

Fluocinolone acetonide (FAc) is a corticosteroid that is available as a small (length 3.5 mm, diameter 0.37 mm), nonbiodegradable, intravitreal implant, designed for injection into the vitreous cavity via the pars plana using a 25-gauge proprietary injector that is approved for the treatment of DME. It releases a steady, low dose of FAc (0.2 μg/day) for up to 36 months [9], thus providing stable, extended control of DME and reducing the economical and clinical burden of repeated injections. As with corticosteroids in general, FAc is thought to reduce retinal edema through its anti-inflammatory effects, by inhibition of phospholipase A2 [10]. In addition, FAc inhibited VEGF secretion and VEGF mRNA expression in vitro, in a human retinal pigment epithelial cell line (ARPE-19) [11]. The efficacy of FAc intravitreal implant in DME was evaluated in two phase 3 studies—FAME A and B [12]. The findings from the FAME studies are supported by a number of real-world studies, mainly conducted in Europe, where the FAc implant is currently indicated for the treatment of vision impairment associated with chronic DME, considered insufficiently responsive to available therapies. Furthermore, there is increasing interest in its potential effect in reducing retinal neurodegeneration and protecting retinal photoreceptors [13].

The aim of our study was to assess, retrospectively, the long-term morpho-functional effect of the FAc implant in patients with chronic DME and treated in our clinic.

## 2. Materials and Methods

This retrospective study was approved by the Ethics Committee/Institutional Review Board of the Catholic University (ID 3166). This research adhered to the tenets of the Declaration of Helsinki and informed consent was obtained from all patients, after a full and detailed explanation of the goals and procedures of the study. All the clinical, imaging, and electrophysiological data reported in this study were retrospectively analyzed.

### 2.1. Subjects

Seventeen eyes of twelve consecutive patients with diabetes mellitus, treated with a FAc implant for chronic DME were retrospectively evaluated. The patients were observed between 12/06/2018 and 31/03/2022 at the Ophthalmology Department of Università Cattolica del Sacro Cuore–Fondazione Policlinico Gemelli IRCCS of Rome–Italy.

Inclusion criteria were: age > 18 years, signed informed consent, a clinical and instrumental diagnosis of chronic DME insufficiently responsive to available treatments.

Exclusion criteria were: refusal to sign the informed consent, elevated intraocular pressure (IOP) and unresponsive to medical treatment, retinal or choroidal disease other than diabetic retinopathy that could affect the central macula.

All the patients, according to the drug SPC (summary of product characteristics) indications, were pseudophakic at the time of the implant, and had already received either panretinal laser photocoagulation and/or intravitreal injections of anti-VEGF agents and/or the dexamethasone implant. All study eyes received an intravitreal FAc implant (ILUVIEN^®^, Alimera Sciences, Inc., Europe Ltd by a single surgeon (AMM) under topical anesthesia; the implant was injected in the inferotemporal quadrant at 3.5 mm posterior to the limbus. The injection procedure was facilitated by rotating the needle clockwise and then anticlockwise to allow a gentle penetration (marines maneuver). Antibiotic drops (azithromycin) were administered 2 times daily for 3 days after the injection.

This study involved a cohort of seven males and five females, aged between 64 and 90 (mean 75.12). Eleven patients were affected by type 2 DM and one patient, who was treated bilaterally, by type 1 DM. Existing therapies included oral therapy (8%), insulin therapy (75%), and combined oral and insulin therapy (17%). The mean disease duration was 28.2 years (range 10–40 years). Eight patients (66.66%) had received panretinal laser photocoagulation before FAc implantation. All the included patients had received previous intravitreal injections of anti-VEGF agents and/or a dexamethasone implant, with a mean number of 7.26 intravitreal injections in the study eye prior to FAc implantation (range 2–21). The mean wash-out period before to injection of the FAc implant was 12.23 months (range 3–34).

All demographic and clinical data are reported in Table 1.

### 2.2. Ophthalmological Examination

All patients underwent a complete ophthalmologic examination at baseline and at follow-up visits. Follow-up visits were scheduled at 3–6 months, 12 months and 18–24 months.

BCVA (best-corrected visual acuity) was assessed using ETDRS charts and expressed in number of letters read, while IOP measurements were performed with a Goldmann applanation tonometer. OCT acquisitions were performed using DRI OCT Triton (Topcon, Inc, Tokyo, Japan). SS-OCT and CMT was automatically assessed from each macular scan. Ganzfeld electroretinograms (Retimax, CSO, Firenze, Italy) were recorded with a specific, published protocol (employed to isolate and analyze the photopic negative response (PhNR) from the single flash cone-mediated responses [14,15,16]. The amplitude of the PhNR and the cone b-wave were measured in each recording session. All the above-mentioned exams were performed at each follow-up visit.

### 2.3. Outcome Measures

The main outcome was mean change in CMT at month 24. Secondary outcomes were changes in CMT and BCVA from baseline to month 3–6, 12, and 18–24, as well as the change in cone electroretinogram measurements (cone b-wave and PhNR) from baseline to month 3–6, 12, and 18–24. Adverse events were also recorded.

### 2.4. Data Registration and Acquisition

Data were extracted from the patients’ medical charts and collected using Microsoft Excel.

### 2.5. Statistical Analysis

Assuming normal distribution, data were analyzed using ANOVA. Given multiple comparisons, a conservative *p*-value < 0.05 was considered as statistically significant. Statistical analysis was performed using Origin, version 6.0

## 3. Results

### 3.1. Effects of the FAc Implant on Retinal Morphology and Function at 24 Months

**Figure 1:**
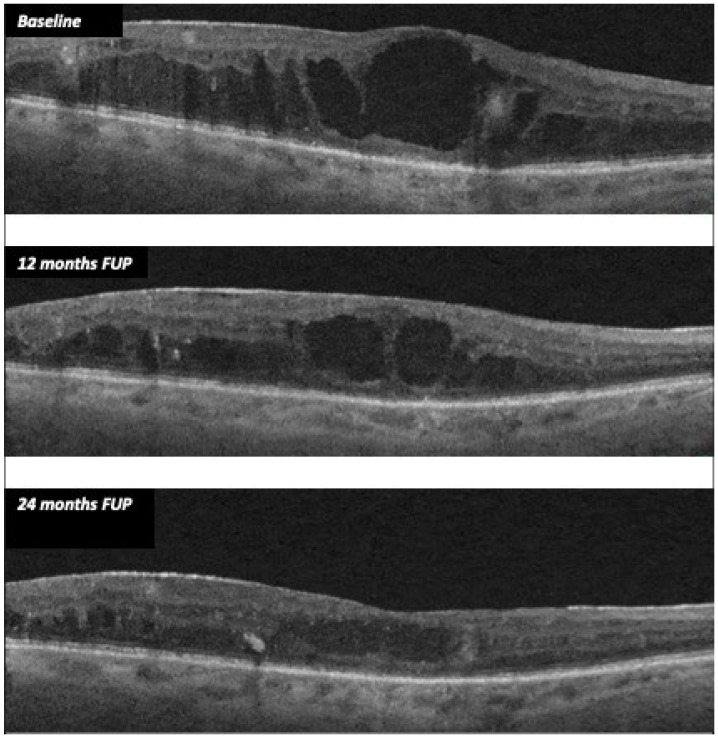
CMT variation during follow-up after FAc implant in case 11.

In treated eyes, we found a significant reduction in CMT, with a stabilization of both BCVA and electrophysiological parameters. Mean CMT decreased from 406.52 µm (±138.74) at baseline to 310 µm (± 130.39) at 24 months (*p* = 0.008), whereas the mean BCVA varied from 55.70 ETDRS letters (±24.07) to 54.58 ETDRS letters (± 23,11) at 24 months (*p* = 0.698). Figure 1 shows CMT variation from baseline to months 12 and 24.

Concerning electrophysiological measurements, PhNR amplitude varied from a mean value of 2.48 µV (± 1.05) at baseline, with a peak time of 61.05 ms (±7.03), to a mean value of 2.41 µV (± 1.03) at 24 months (*p* = 0.797), with a peak time of 61.61 ms (±6.31) (*p* = 0.8). B-wave amplitude showed a reduction from a mean value of 9.44 µV (± 5.65) at baseline, with a peak time of 39.46 ms (± 3.70) to a mean value of 7.51 µV (± 3.70) with a peak time of 37.61 ms (±10.23), albeit the results were not statistically significant (*p* = 0.164). Age-matched healthy controls showed a mean B wave amplitude of 24 µV (SD 6.5) and a mean PhNR amplitude of 7 µV (SD 6.5).

IOP showed a variation from a mean value of 15.11 mmHg (±2.91) at baseline to a mean value of 17.05 mmHg (±6.2) at 24 months (*p* = 0.16). Ophthalmological measurements are reported in detail for each patient in Table 2.

Five patients (41%) underwent bilateral implant, while seven patients (59%) underwent monolateral implant. Ophthalmological data from non-treated eyes were collected for comparison. Among non-treated eyes, mean CMT varied from 293.33 µm at baseline to a mean value of 237.8 µm at 24 months. Mean BCVA changed from 58.5 ETDRS letters at baseline to a mean value of 55.75 ETDRS letters at 24 months. Mean PhNR amplitude varied from 3.61 µV at baseline, with a peak time of 63.40 ms, to a mean value of 3.49 µV, with a peak time of 60.41 ms at 24 months. Mean b-wave amplitude changed from 9.17 µV at baseline, with a peak time of 37.49 ms, to a mean value of 13.31 µV, with a peak time of 38.14 ms at 24 months.

### 3.2. Correlations among Morphological and Functional Parameters at Baseline and End of Follow-Up

**Figure 2:**
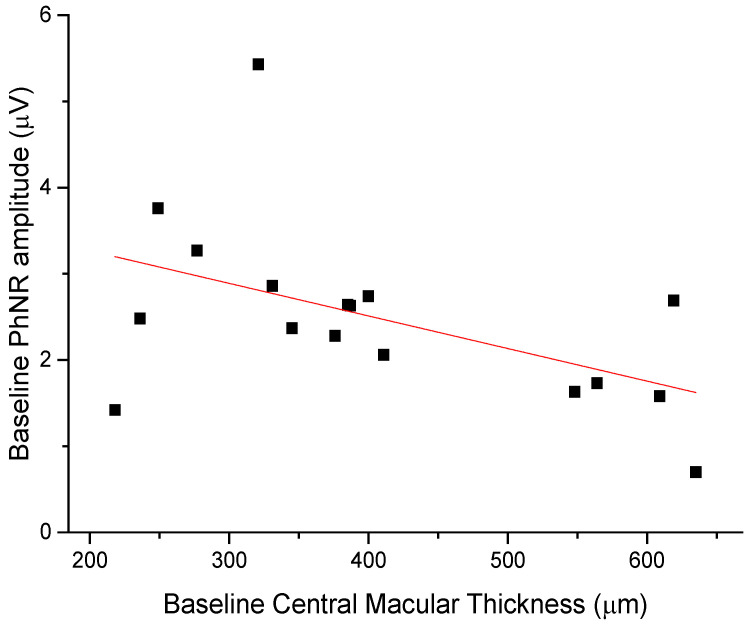
Baseline PhNR amplitude was negatively correlated with CMT (r = −0.7, *p* < 0.01).

**Figure 3:**
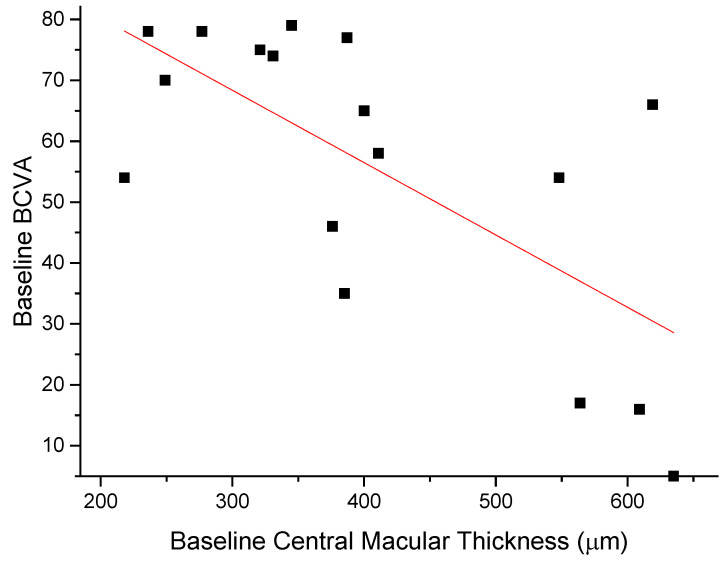
Baseline BCVA was negatively correlated with CMT (r = −0.5, *p* < 0.05).

**Figure 4:**
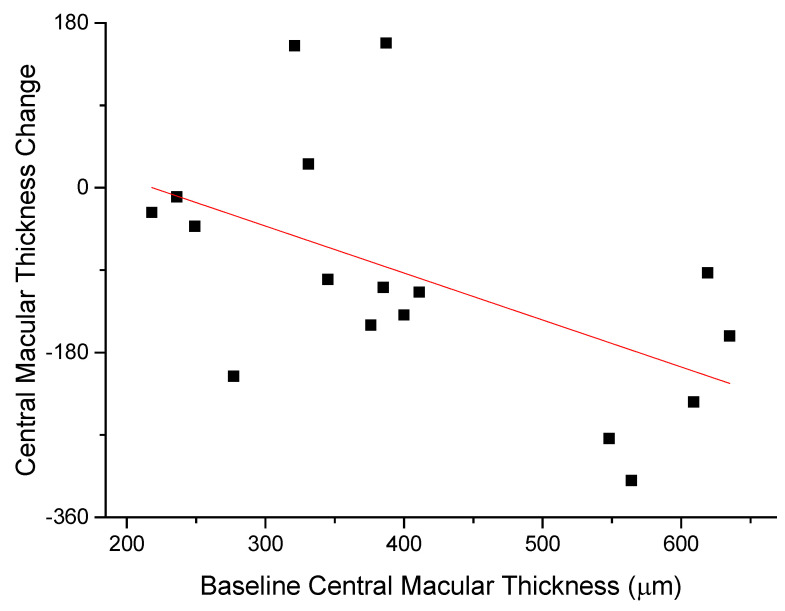
At the end of follow-up, the change in CMT was negatively correlated with baseline CMT (r= -0.53, *p* = 0.03).

**Figure 5:**
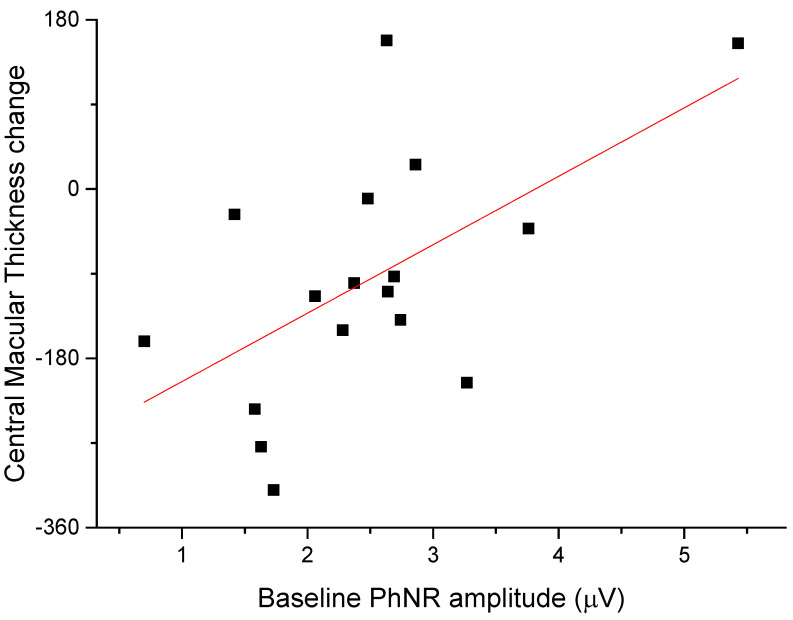
At the end of follow-up, the change in CMT was positively correlated with baseline PhNR amplitude (r = 0.58, *p* < 0.01).

The anti-edema effect tended to be stronger in patients with the poorest baseline retinal morphology (CMT) and function (PhNR). At baseline, PhNR amplitude and BCVA were negatively correlated with CMT (see Figure 2 and Figure 3; r = −0.7, *p* < 0.01; r = −0.5, *p* < 0.05, respectively). At the end of follow-up, the change in CMT was negatively correlated with baseline CMT (Figure 4, r= −0.53, *p* = 0.03) and positively with baseline PhNR amplitude (Figure 5, r = 0.58, *p* < 0.01).

## 4. Discussion

The aim of the present study was to assess the morphological and functional effects of the intravitreal FAc implant in patients with chronic DME over a period of 24 months. As well as these effects, correlations between morphological and functional parameters were also evaluated. At 24 months, our data showed a statistically significant reduction in CMT and the stabilization of both visual acuity and photopic ERG b wave PhNR amplitudes. Both BCVA and PhNR amplitudes tended to decrease as CMT increased. The change in CMT recorded at the end of follow-up related to baseline CMT and PhNR amplitude values, showing the best outcomes in patients with worse baseline morphology (CMT) and function (PhNR) values.

The long-term morphological effectiveness of the FAc implant has been demonstrated in several studies. Bailey et al. (2017) [17] found a reduction in CSFT from 451.2 to 355.5 μm after 24 months. The follow-up analysis by Bailey et al. (2021) [18] showed that this effect, along with a reduction in macular volume, was evident after 3 years of therapy. The effectiveness of FAc on DME can be explained considering that steroids inhibit proinflammatory mediators and change the local ratio of laminin isoforms in the endothelial basal membrane, improving the blood–retinal barrier and limiting permeability and leakage by strengthening capillaries tight junctions [19].

The effect of the FAc implant on BCVA has been widely described with several retrospective studies reporting maintenance or improvement in visual acuity lasting up to 24 months. Furthermore, it has been reported that > 15% of eyes show an improvement in BCVA of 15 letters during 3–18 months [17]. Analogously, prospective studies showed functional effectiveness of FAc implant in terms of BCVA [20].

The improvement of ERG parameters found in the present study could be related directly to the intravitreal FAc or indirectly to the decrease in CMT. However, CMT reduction is localized to the macula, which contributes only partially to the full-field ERG response. The effect of the drug is most likely to influence the ERG changes. This result support the hypothesis of a neuroprotective effect of FAc in human retinas, as previously postulated in preclinical [13] and clinical studies [21,22]. Preclinical studies conducted by Glybina et al. had investigated the neuroprotective properties of low-dose, sustained-release intravitreal FAc in transgenic S334ter-4 rats [13]. They found ERG amplitudes reduced in the control groups, whereas in the FAc-treated groups no statistically significant attenuation of the ERG amplitudes was observed at 9 weeks. In addition, the histologic evaluation demonstrated that, in the FAc-treated groups, the retinal outer nuclear layer (ONL) thickness was greater than in the control groups. When counting microglial cells, the FAc-treated groups presented fewer activated and number of microglial cells in the photoreceptor cell layer. These studies suggest that sustained release of FAc may reduce retinal degeneration and protect retinal photoreceptors. Clinical studies conducted by Lynch et al. hypothesized that the intravitreal FAc implant may affect the rate of DRN (diabetic retinal neurodegeneration) in patients with persistent DME, decelerating the rate of inner retinal thinning [21]. Analogously, Pessoa et al. found no evident retinal neurodegeneration in the 2-year period following treatment with FAc in vitrectomised and non-vitrectomised diabetic eyes with DME [22]. In accordance with these literature data, in a previous study analyzing 18 chronic DME eyes treated with FAc, we found an improvement in mean PhNR amplitude from 2.76 (1.65) µV at baseline to 3.73 (2.32) µV at month 1–3 (mean difference 0.91 (1.14) µV, 95% CI ± 0.54 µV, *p* = 0.003) and an improvement of the b-wave amplitude from 8.83 (4.52) µV at baseline to 10.05 (5.04) µV at month 1–3 (mean difference 1.22 (2.23) µV, 95% CI ± 1.08 µV, *p* = 0.0384). These ERG positive changes tended to endure up to months 9–14, although they did not reach statistical significance after month 3 [23]. These results supported the hypothesis that intravitreal FAc implant may exert a protective retinal effect in patients with DME and encouraged us to investigate the long-term outcomes.

To the best of our knowledge, no previous studies have investigated the relationship between morphology, measured by CMT, and retinal function, assessed by cone ERG in eyes treated with FAc. Worse PhNR recorded at baseline predicted a better outcome of the main study variable (CMT) at the end of follow-up. PhNR is the negative component of the signal that is highly sensitive to ischemic/metabolic perturbation of the inner retina. DME is the expression of inner retina pathology [24] and appears to be correlated with the PhNR amplitude loss.

The majority of patients underwent bilateral FAc implantation. In five patients who received a monolateral implant, the fellow untreated eye served as a test control. In four out of five untreated eyes (80%), PhNR showed a severe reduction from baseline to month 24, confirming a progressive deterioration with the progression of DR.

The lack of a consistent improvement of BCVA and PhNR in the study sample could be influenced also by a selection bias. All patients included in the study had long-term DME, thus explaining the fact that the improvements in CMT were not reflected in BCVA and PhNR. However, we hypothesize that without FAc implant there would be a deterioration of these parameters, as demonstrated in the untreated fellow eyes. Hence, we acknowledge that the relationship between the gain in BCVA and the decrease in CMT needs to be further investigated in a cohort of diabetic patients with short-term DME versus the current study cohort.

A limitation of the study is the small sample size and the heterogeneity of the study population, which included both patients with PDR and patients with NPDR, as well as patients with different duration of DME and different pre-FAc therapies and wash-out periods. Further studies with larger sample size and more homogeneous population would be needed to support the findings of the present study.

## 5. Conclusions

In conclusion, the results of this retrospective long-term study indicate that a significant reduction in CMT was found in DME patients following therapy with the FAc implant. Retinal morphology and function at baseline significantly predicted CMT changes observed at month 24. Such correlations might help to better characterize the patients who may benefit from this treatment.

## Figures and Tables

**Table 1:** Demographic and clinical data.

	Age at Baseline (Years)	Type of Diabetes	Duration of Diabetes (Years)	Therapy	Type of DR	Previous PRP (YES/NO)	Washout Period from Previous IVI before FAc (Months)
**Case 1**	74	II	24	Insulin	NPDR	YES	4
**Case 2**	79	II	28	Insulin	NPDR	YES	5
**Case 3**	80	II	28	Insulin	PDR	YES	9
**Case 4**	70	II	40	Insulin	PDR	YES	23
**Case 5**	70	II	40	Insulin	PDR	YES	26
**Case 6**	70	II	29	Insulin	PDR	YES	12
**Case 7**	73	II	10	Insulin + Linagliptin	NPDR	YES	6
**Case 8**	73	II	10	Insulin + Linagliptin	NPDR	YES	7
**Case 9**	77	II	40	Insulin	NPDR	YES	6
**Case 10**	87	I	35	Insulin	NPDR	YES	34
**Case 11**	87	I	35	Insulin	NPDR	YES	26
**Case 12**	90	II	15	Metformin	NPDR	NO	2
**Case 13**	71	II	30	Insulin	NPDR	YES	8
**Case 14**	72	II	30	Insulin	NPDR	YES	15
**Case 15**	64	II	33	Insulin + Metformin	PDR	NO	3
**Case 16**	74	II	25	Insulin	PDR	YES	9
**Case 17**	66	II	30	Insulin	NPDR	YES	13

DR diabetic retinopathy, IVI intravitreal injection, NPDR, non-proliferative diabetic retinopathy, PDR proliferative diabetic retinopathy, PRP panretinal photocoagulation.

**Table 2:** Ophthalmological data.

PN	CMT (µm)	BCVA (ETDRS)	PhNR Amplitude (µV)	B-Wave Amplitude (µV)	IOP (mmHg)
Case 1					
Baseline	387	77	2.63	12.25	10
3–6 months	425	64	4.20	15.97	13
12 months	624	64	4.15	14.45	17
24 months	545	65	3.87	12.87	15
Case 2					
Baseline	411	58	2.06	2.99	11
3–6 months	331	70	1.94	4.77	18
12 months	314	65	2.05	3.58	12
24 months	297	64	1.63	2.96	15
Case 3					
Baseline	376	46	2.28	5.53	12
3–6 months	219	35	2.85	10.12	10
12 months	233	47	2.01	6.95	14
24 months	233	48	1.3	4.06	15
Case 4					
Baseline	345	79	2.37	12.05	18
3–6 months	296	70	4.82	14.36	18
12 months	272	65	4.10	10.80	17
24 months	245	77	3.45	7.97	32
Case 5					
Baseline	249	70	3.76	15.93	19
3–6 months	215	72	4.73	12.38	19
12 months	207	62	3.10	10.38	17
24 months	207	74	1.70	8.40	34
Case 6					
Baseline	635	5	0.70	6.46	14
3–6 months	509	35	0.80	6.02	19
12 months	548	40	0.94	4.37	15
24 months	473	28	1.35	7.70	13.7
Case 7					
Baseline	277	78	3.27	7.82	16
3–6 months	218	78	3.12	7.20	14
12 months	232	78	2.64	7.19	15
24 months	271	76	2.14	7.16	15
Case 8					
Baseline	236	78	2.48	6.28	14
3–6 months	235	78	1.89	8.17	15
12 months	231	78	1.87	8.14	15
24 months	226	77	1.87	8.1	15
Case 9					
Baseline	218	54	1.42	3.59	16
3–6 months	204	60	1.82	3.85	16
12 months	197	67	2.31	4.32	16
24 months	191	65	1.58	3.99	16
Case 10					
Baseline	400	65	2.74	9.85	18
3–6 months	256	60	4.18	9.62	16
12 months	261	64	3.44	6.31	16
24 months	261	64	1.1	5.27	14
Case 11					
Baseline	609	16	1.58	5.84	16
3–6 months	543	17	2.57	8.02	17
12 months	511	18	1.68	5.33	15
24 months	375	10	3.09	6.32	13
Case 12					
Baseline	321	75	5.43	15.28	16
3–6 months	349	69	4.96	18.48	12
12 months	356	69	4.54	17.01	17
24 months	476	57	3.72	16.99	17
Case 13					
Baseline	385	35	2.64	8.52	13
3–6 months	281	50	1.61	7.79	13
12 months	253	46	0.74	6.92	16
24 months	276	19	2.64	7.48	15
Case 14					
Baseline	548	54	1.63	4.70	14
3–6 months	263	49	0.55	9.28	21
12 months	275	35	1.15	6.37	11
24 months	274	32	1.15	6.37	11
Case 15					
B Baseline	619	66	2.69	12.73	19
3–6 months	490	70	2.69	14.40	18
12 months	552	74	7.45	17.08	14
24 months	526	75	3.18	12,99	19
Case 16					
Baseline	564	17	1.73	5.62	19
3–6 months	386	21	4.29	5.39	18
12 months	368	21	7.86	7.66	17
24 months	244	28	4.00	4.20	14.3
Case 17					
Baseline	331	74	2.86	25.12	12
3–6 months	314	82	3.31	4.88	15
12 months	335	80	3.34	4.97	16
24 months	357	77	3.19	4.41	15

BCVA best-corrected visual acuity, CMT central macular thickness, ETDRS early treatment diabetic retinopathy study, IOP intraocular pressure, PhNR photopic negative response.

## Data Availability

Data available from authors.
